# Perfluorophenyl Ether and Related Polymers^1^

**DOI:** 10.6028/jres.068A.025

**Published:** 1964-06-01

**Authors:** Walter J. Pummer, Leo A. Wall

## Abstract

The syntheses of perfluorophenyl ether and poly (perfluorophenylene ethers) are described. These materials were prepared by the reaction between potassium pentafluorophenoxide and hexafluorobenzene in different solvents with varying conditions of temperature and pressure. In dimethylformamide or tetrahydrofuran, potassium pentafluorophenoxide shows little tendency to react with hexafluorobenzene. In methanol, only exchange products such as pentafluoroanisole were observed. No polymers were obtained in these solvents. In water, the reactivity of pentafluorophenoxide salts was increased greatly, for reaction occurs readily with or without the presence of hexafluorobenzene. The reactions in aqueous systems are complex and give rise to a variety of products. From these reactions the following compounds were isolated and identified: perfluorophenyl ether, bis(pentafluorophenoxy)tetrafluorobenzene, 2,4,5,6-tetrafluororesorcinol, pentafluorophenoxytetrafluorophenol, and various polyperfluorophenylene ethers of varying chain lengths. Identification of the smaller molecules was made by chemical, mass spectrometer, infrared, and nuclear magnetic resonance analyses. Poly(perfluorophenylene ethers) were prepared having molecular weights of 1700, 4300, 6500, and 12,500. A crosslinked polyether of this type was also synthesized, which had rubbery characteristics above 90 °C.

## 1. Introduction

The synthesis of perfluorophenyl ether has been the object of research by investigators in the aromatic fluorine field for many years. From this compound, thermal stability data could be obtained which may be applicable to polyfluorophenylene ethers. Interest in this compound developed because polyperfluoroplienyl [1]^2^ synthezised in this laboratory had some undesirable physical properties, such as brittleness and insolubility. These properties could possibly be improved by the incorporation of some atom or groups of atoms between the phenyl rings to introduce bond angles other than 180°. Since aryl ethers are among the more stable organic compounds [2, 3], it appeared likely that the poly(perfiuorophenylene ethers) (PPPE) may have good thermal stability as well as improved physical characteristics.

Partially fluorinated diphenyl ethers have been prepared by Ullman reactions involving phenolic salts and a halobenzene with copper catalysts at high temperatures in sealed tubes. By this method, 3(trifluoromethyl)phenyl ether [4] and pentafluorophenyl phenyl ether [5] were synthesized. Still other types of fluorophenyl ethers [6, 7] were obtained as byproducts from various nucleophilic reactions.

Some polymers of fluoroaromatic ethers have also been reported. Poly (perfluorophenylene ether) [8] and polyperfluorophenylene thioether [9] were obtained by thermal decomposition of sodium pentafluorophenoxide and potassium pentafluorothiophenol, respectively. Haszeldine [10] has also reported a polyfluoro ether obtained from 2,3,4,5,6-pentafluorobenzyl alcohol.

In an earlier report [5] we disclosed the first synthesis of perfluorophenyl ether. The present article describes some other syntheses of this compound as well as the pertinent details relating to its structure and identification.

## 2. Pentafluorophenol

Since most of the projected syntheses of perfluorophenyl ether involved the use of pentafluorophenol or its salts, an improved simple process was needed for the synthesis of pentafluorophenol. This intermediate was prepared previously [11–13], however in these methods separation problems were often encountered. The reactions of aqueous ammonia or alkyl amines with hexafluorobenzene gave excellent yields of the various substituted pentafluoroanilines [5]. On this basis, it appeared that an aqueous medium may also be useful for the preparation of pentafluorophenol. An attempt to synthesize pentafluorophenol in aqueous sodium hydroxide from hexafluorobenzene was unsuccessful [13], but aqueous potassium hydroxide [5] and hexafluorobenzene gave consistently good results. The best results [5] were obtained by employing a slight excess over the stoichimetric amount of hexafluorobenzene in relation to the base used, with the reaction conducted in a closed pressure vessel at 175 °C for 5 hr. The potassium pentafluorophenoxide, precipitated upon cooling, is usually pure enough for subsequent reactions. The yields range between 80 and 85 percent.

Various catalysts such as iron filings, copper powder, or copper oxide cause the reaction to occur as low as 120 °C, but the conversion is lower and longer reaction times are required. In the presence of excess base, high temperature (190 °C), and long-reaction times (20 hr), only carbonlike solids were obtained; at 150 °C for 5 hr only slight indication of reaction was observed and 77 percent of the hexafluorobenzene was recovered.

From this latter reaction, however, there was isolated a new tetrafluorodihydroxybenzene. This compound was distillable without decomposition and solidified upon cooling (mp 95 to 96 °C). It was water soluble and gave the characteristic blue ferric chloride test for phenols. Chemical and mass spectrometer (parent peak at 182 mass units) analyses furnished further evidence as to its structure. The near infrared spectra in carbon tetrachloride solution showed the hydroxyl band at 3570 cm^−1^, which is characteristic of these fluorophenols (see table 1, B). It is of interest to note that this compound does not exhibit the split hydroxyl band in this region, which may have been indicative of the ortho isomer [5]. In the 1100–1350 cm^−1^ region, the C–F absorption occurs at 1105 cm^−1^ and again at 1300 cm^−1^. Definite assignment of other structural characteristics becomes uncertain in this region because of the C–F absorption even though aromatic fluorine absorption is less in this region than the aliphatic. However, by comparison with some other spectra of fluorine-containing compounds, the band at 1235 cm^−1^ region is peculiar to phenolic-type compounds and may be due to the OH deformation vibration [16]. The para isomer, i.e., 2,3,5,6-tetrafluorohydroquinone, has been synthesized previously [14, 15], but its melting point is much higher (168 to 169 °C). Therefore our compound could only be the ortho or meta isomer. Examination by nuclear magnetic resonance spectra has definitely eliminated the ortho isomer. Therefore, the compound isolated from the above reaction must be tetrafluororesorcinol.

## 3. Perfluorophenyl Ether

Application of the procedures [5] by which the mixed fluoroaryl ethers were prepared, to the synthesis of perfluorophenyl ether, met with partial success. Unlike potassium phenoxide, which reacts with hexafluorobenzene in *N*,*N*-dimethylformamide at room temperature, potassium pentafluorophenoxide fails to react under these conditions. By refluxing for 14 hr, a 16 percent yield of perfluorophenyl ether was obtained [5]. These examples also exemplify the differences in reactivity between the phenoxide and pentafluorophenoxide ion, the former being a stronger nucleophile because even at room temperature some of the 1,4-diphenoxy-2,3,5,6-tetrafluorobenzene was isolated. In the above reactions hydrated phenoxide salts were used. Attempts to react potassium pentafluorophenoxide with hexafluorobenzene in dimethylformamide, using anhydrous conditions and refluxing, resulted in the formation of pentafluoro-*N,N*-dimethylaniline. Apparently under this basic condition, cleavage of *N*,*N*-dimethylforrnamide occurs, liberating dimethylamine. The product obtained was confirmed by comparison with an authentic sample prepared by other means [5].

In tetrahydrofuran, no improvement in the yield of perfluorophenyl ether from hexafluorobenzene and potassium pentafluorophenoxide was observed, even though the reaction was carried out in a bomb at 120 °C for 18 hr.

When methanol was used as the reaction medium, pentafluoroanisole was obtained in a fair yield. Although the acid-base reaction (eq 1b)

$$C_{6}F_{5}\text{OK}+{\text{CH}}_{3}\text{OH}\overset{a}{\underset{b}{⇄}}\underset{\begin{array}{c} c↓C_{6}F_{6}\\ C_{6}F_{5}{\text{OCH}}_{3}+\text{KF} \end{array}}{{\text{CH}}_{3}\text{OK}}+C_{6}F_{5}\text{OH}$$ (1)

should be the preferred reaction, the driving force of the reaction appears to be replacement of fluorine (eq 1c) in hexafluorobenzene by methoxide ion. Reactions of this type have been shown [11, 12] to occur quite rapidly.

## 4. Properties of Perfluorophenyl Ether

The perfluorophenyl ether [5] was usually isolated by diluting the reaction mixture with water and separating the fluorocarbon layer. After removal of the unreacted hexafluorobenzene by distillation, the residue solidified upon cooling. The initial fraction obtained from the sublimation of this residue was a white crystalline solid, mp 68–69 °C. The high melting point of this compound was unusual, as it was expected to be similar to that of phenyl ether, mp 27 °C. Chemical analysis indicated perfluorophenyl ether. The compound contained only carbon, fluorine, and oxygen. Analysis for hydrogen was negative. However, other verification of the structure was required since there are only small percentage differences of the elements in two- and three-ring compounds in this series. Confirmatory evidence was obtained from mass spectrometric analysis, which showed the parent peak at 350 mass units. Vapor-phase chromatographic analysis on a Viton-A column at 200 °C indicated an expected retention time slightly less than that of pentafluorophenyl phenyl ether.

The infrared spectra showed the characteristic ring vibration at 1520 cm^−1^. The aryl ether band is usually found in the 1270–1230 cm^−1^ region [17], but in some cases, it may be as low as 1218 ±10 cm^−1^ [18]. In perfluorophenyl ether there is a single strong band at 1170 cm^−1^ (table 1, E). This band appears in all spectra of the limited number of compounds investigated containing the C_6_F_5_—O—R ether grouping (C to G in table 1). It is of interest to note that the fluorophenols do not show this band. Similarly, the fluoroaryl ethers do not exhibit the band at 1235 cm^−1^. However, both bands (1170 cm^−1^ and 1235 cm^−1^) are shown by pentafluorophenoxy-tetrafluorophenol (table 1, C). In pentafluorophenyl phenyl ether (table 1, F) two distinct bands appear. The one at 1205 cm^−1^ is probably associated with the phenyl ether linkage (C_6_H_5_—O—R), whereas the other at 1169 cm^−1^ seems to be the pentafluorophenyl ether band. These same two bands appear in the spectra of 1,4-diphenoxytetrafluorobenzene. The bands in all of these compounds near 1100 cm^−1^ and 1300 cm^−1^ are probably carbon fluorine vibrations. Although there is some variance in the 1100 cm^−1^ region, the one at 1300 cm^−1^ remains fairly constant. Comparison bands in this 1100 cm^−1^ to 1300 cm^−1^ region are also listed for hexafluorobenzene and perfluorobiphenyl (table 1, H and I).

## 5. Reactions of Pentafluorophenoxide Salts in Water

The low reactivity of pentafluorophenoxide salts in *N,N*-dimethylformamide, tetrahydrofuran, and methanol, although they are soluble in these solvents, led to investigations in other media in which the reactivity of the salt could be enhanced. The excellent yields of pentafluorophenol and pentafluoroaniline obtained in aqueous medium, even though the reactions are heterogeneous in nature, suggested that water may be a useful reaction solvent for preparing the fluorophenylene ethers. Reactions performed in water would also yield important information on the hydrolytic stability of the ring fluorines as well as the ether linkages.

Hexafluorobenzene is stable to hydrolysis by water in the absence of base to at least 300 °C (limit of temperature range of equipment used), but as reported earlier in this article, in the presence of base, reaction can occur at a temperature as low as 120 °C in a closed system under autogenous pressure. Thus, it appeared feasible to consider the preparation of polyfluoroetliers directly from hexafluorobenzene. In the first step, therefore, the usual conditions for preparing pentafluorophenol from hexafluorobenzene and potassium hydroxide were maintained (eq 2a), and after 5 hr the temperature was raised to

$$C_{6}F_{6}+2\text{KOH}+H_{2}O\frac{a}{175{}^{\circ}C}\to \underset{\begin{array}{c} b↓225{}^{\circ}C\\ \text{products} \end{array}}{C_{6}F_{5}\text{OK}}+{\text{KF+H}}_{2}O$$ (2)

225 °C for an additional 15 hr (eq 2b). The *p*H of the solution was then only slightly alkaline to alk-acid test paper, indicating almost complete reaction of the base. The experiment was designed to produce poly(perfhiorophenylene ethers) in which there was no hydroxyl end group (eq 3),

$$xC_{6}F_{5}\text{OK}+C_{6}F_{6}\to C_{6}F_{5}O{\left(C_{6}F_{4}O\right)}_{x}C_{6}F_{5}+x\text{KF}$$ (3)

such as occurs in the thermal decomposition of sodium pentafluorophenoxide (eq 4).

$$xC_{6}F_{5}\text{ONa}\frac{\;}{345{}^{\circ}C}\to C_{6}F_{5}({\text{OC}}_{6}F_{4})x\text{ONa}+\text{other products}$$ (4)

However, the reaction was observed to be rather complex in nature. Partial separation of the products was obtained by vacuum distillation. The volatile fraction solidified on cooling, mp 83 to 87 °C. This fraction was subdivided further into neutral and acid components by extraction with aqueous sodium carbonate. The neutral fraction was mainly perfluorophenyl ether, along with a trace of bis(pentafluorophenoxy)-tetrafluorobenzene, which were separated by vacuum sublimation. The perfluorophenyl ether obtained from this reaction was identical to the one prepared previously. The base-soluble component was shown to be pentafluorophenoxytetrafluorophenol by chemical and mass spectrometric (parent peak at 348 mass units) analyses. The nearinfrared spectra of this compound showed the single hydroxyl band at 3570 cm^−1^, similar to other fluorophenols [5]. In the 1300 cm^−1^ to 1100 cm^−1^ region the compound showed both the ether band at 1171 cm^−1^ as well as the band due to the hydroxyl at 1237 cm^−1^ (table 1, C). This hydroxy ether was insoluble in water and failed to give a positive test for phenol in aqueous ferric chloride; however, a positive test can be obtained by the addition of ethanol.

From the nonvolatile fraction a green-tinted grease was obtained by sublimation; it showed a hydroxyl vibration in the near-infrared region. After removal of this material and upon cooling, the residue was a dark-brown brittle glass. Both compounds appear to be low molecular weight polyfluoroethers; they were not further investigated, except to observe that the brown glass was insoluble in benzene after this thermal treatment.

Attempts were made to increase the yield of perfluorophenyl ether by employing a large excess of hexafluorobenzene in relation to base, but a reaction in which a 16:1 ratio of hexafluorobenzene to potassium pentafluorophenoxide was used actually gave much smaller yield.

A complicating feature of this reaction is the fact that potassium pentafluorophenoxide alone in water under these conditions will react to produce tarry products. From this reaction pentafluorophenoxytetrafluorophenol was isolated. Althouth there is a slight discrepancy in melting points between this compound and the hydroxy ether obtained when hexafluorobenzene was used (no melting point depression on admixture), the infrared spectra of the two compounds were almost identical, with similar bands in the 1300 cm^−1^ to 1100 cm^−1^ region (table 1, C).

## 6. Reaction of Pentafluorophenoxide Salts in Pentafluorophenol, Poly(Perfluorophenylene Ethers)

This reaction was primarily designed to produce pentafluorophenoxytetrafluorophenol for comparison purposes with those compounds isolated previously. However, none of this compound was obtained; only polymeric material was formed. The main product was an amber-colored, tacky material (I), soluble in most organic solvents. The material was divided into two fractions (II and III) by dissolving the polymer in methanol and using water as a precipitant.

The precipitate (II), after drying in vacuo, was a clear light-brown gum which flowed freely on slight warming. The infrared spectrum of this fraction (II) (table 1, D) showed it to be a fluorophenylene ether containing a free hydroxyl group, probably as an end group. This spectrum was similar to those of pentafluorophenoxytetrafluorophenol (table 1, C). However, in the spectrum of fraction II, the relative intensity of the free hydroxyl group was very much less than that of the broad pentafluorophenyl ether band at 1170 cm^−1^, indicating a polyfluorophenylene structure. No attempt was made to correlate these relative intensities with the molecular weight of the polymers.

**Figure 1:**
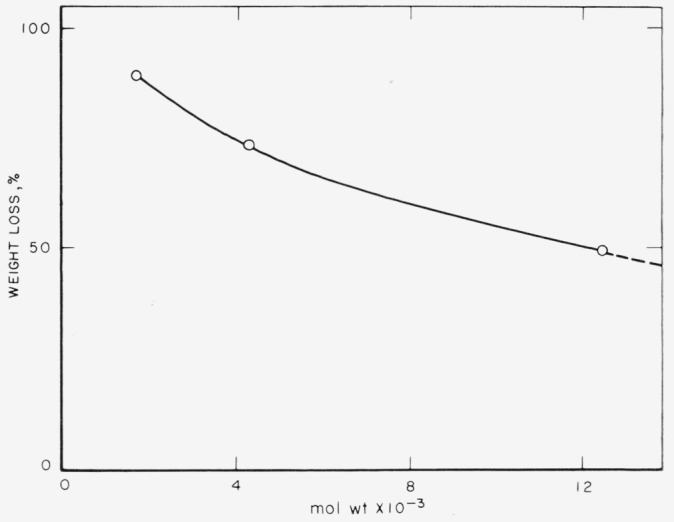
Pyrolysis of poly (perfluorophenylene ethers) percent weight loss upon heating for ½ hr at 400 °C in a vacuum. Result for crosslinked sample, ––→.

The molecular weight of fraction II was found to be 1700, as determined by vapor-pressure osmometry. The average chain length was ten units, assuming the repeating unit to be—C_6_F_4_O—(formula weight 164). From pyrolysis data the polymer appeared to be a mixture of mers with varying chain lengths; 60 percent of the material sublimed as a white solid after 0.5 hours at 250 °C in vacuo and only 10 percent residue remained after 0.5 hours at 400 °C (see fig. 1).

Since the composition of the poly(perfluorophenylene ether) (II) was typical of condensation polymerization, several methods could be used to increase the molecular weight of the polymer. In one attempt potassium pentafluorophenoxide was added to a sample of the nonfractionated material (I) and the mixture was heated at 150 °C for 2 hours. The dark-brown, brittle glass obtained was still methanol soluble. The molecular weight was 1800; apparently the additional phenoxide ion reacts with itself or smaller chain molecules rather than adding onto longer existing chains.

One of the difficulties of condensation polymerization is the combining of the smaller chain units produced into one large polymeric unit. Since the poly (fluorophenylene ethers) isolated, as described above, all show a free hydroxyl group in their infrared spectra, a method for combining chains may be found which utilizes this hydroxyl group in the form of the potassium salt. The procedure involved was similar to other melt polymerization techniques [19]. Fraction III (methanol soluble) was converted to the potassium salt and heated for 0.5 hour at 175 °C and then at 200 °C. By this procedure a fraction was obtained which was benzene soluble but methanol insoluble. It was a light straw-colored glass, somewhat brittle, and had a low softening point (60–80 °C). The molecular weight of this material was 4300; it contained approximately 26 phenyl units. The infrared spectrum showed the polyfluorophenylene ether band at 1170 cm^−1^. Since this fraction was still in the salt form, the hydroxyl bands were absent in their respective regions. Pyrolysis at 400 °C for 0.5 hour in vacuo volatilized 72.4 percent of the polymer. Of the total volatiles obtained, only 7.1 percent was volatile at room temperature. Since the polymer was not preheated before raising the temperature to 400 °C, this 7.1 percent fraction could be traces of solvent or low molecular weight material occluded in the polymer.

A higher molecular weight poly (perfluorophenylene ether) (mol wt 6500; average of 40 phenyl units) was obtained by simply recycling the above polymer (mol wt 4300) through the melt process. The polymer was still light colored and not so brittle. Very little decomposition occurred during the heating cycles. The amount of branching in the 4300 or 6500 molecular weight fractions was not determined.

A poly (perfluorophenylene ether) of 12,500 mol wt was obtained by heating excess potassium pentafluorophenoxide and pentafluorophenol at 200 °C for 72 hours. The polymer was a dark-brown glass which had a softening point of 80 °C and flowed freely at 100 °C. Pyrolysis at 400 °C for 0.5 hour gave a 50 percent weight loss. Heating at 260 °C for 18 hours appeared to crosslink this polymer; the product obtained was a hard brittle glass, insoluble in organic solvents. The polymer softened at 90 °C and possessed rubbery characteristics at temperatures up to 300 °C. Pyrolysis at 400 °C for 0.5 hour gave a 47 percent weight loss. After this treatment the specimen was smaller in size, but retained its original shape. The polymer itself is being evaluated as a potential high temperature rubber. The pyrolysis data of these polymers are shown in figure 1. The molecular weight of the crosslinked polymer is unknown, but from the weight loss we can obtain the general shape of the curve. The leveling off of the curve is interpreted as the breaking of phenyl to oxygen links, so that the thermal stability of these perfluorophenylene ethers is not much greater than 400 °C.

Some physical properties of these polyperfluorophenylene ethers are summarized in table 2. Two factors become apparent from the table. First, the softening point increases as the molecular weight increases. The range indicates where softening begins and complete melting occurs. For the cross-linked polymer the figure represents the point at which rubbery behavior appears. At no temperature to 400 °C does this polymer “puddle.”

The other point of interest is the change in solubility with increasing molecular weight. From this solubility behavior it is possible to obtain a qualitative estimate of the molecular weight of the polymers obtained from various experiments. For example, the 12,500 mol wt fraction is insoluble in ether whereas the lower molecular weight polymers are soluble.

The crosslinked polymer appears to be of the most interest. The nature of these crosslinks is obscure at the moment. At first it was believed that these links were caused by reaction of the polymer with the glass walls of the reaction vessel in which it was made, i.e., by stripping fluorine between the rings. However, it was recently reported [20] that the hydrocarbon phenoxylenes also crosslinked but at a much lower temperature (150 °C), so that the removal of a mole of fluorine does not appear to be an integral part of the crosslinking mechanisms. Also, Halpern [21] has also shown that 2,6-dimethylphenylene oxide polymers crosslink independent of the molecular weight of the polymer sample used, at 500 °F. The amount of branching in the crosslinked ether has not been determined; and from the carbon analysis of the polymer, branching does not appear to be extensive. The carbon analysis is compatible with a large cyclic structure, and it could occur by coupling remaining—OK end groups with a phenyl ring somewhere along the interior of the chain. Although the potassium residue remaining in the crosslinked polymer has not changed very much from that of the 12,500 mol wt fraction, it is difficult to determine at this stage whether this group,—OK, is still part of an end group, or whether because of the insolubility of the system, this residue is occluded in the polymer matrix. Perhaps, a better insight into the nature of these crosslinks will become apparent from an examination of the volatiles obtained from the pyrolysis of the polymers. These products are currently under investigation.

## 7. Discussion

It is evident that poly (perfluorophenylene ethers) are easier to obtain than the simple ether itself. This fact demonstrates that perfluorophenyl ether is a reactive intermediate, even with a weak nucleophile, such as pentafluorophenoxide ion, especially in aqueous systems. Tatlow [9] reported similar results in attempts to prepare perfluorophenyl thioether in pyridine, only polymers being formed. These fluorophenylene sulfide polymers are essentially para linked and high melting. Proof of the para structure was obtained by desulfurization with Raney nickel, which led to the isolation of pentafluorobenzene and 1,2,4,5-tetrafluorobenzene. In the case of polyperfluorophenylene ethers, structure determinations are more difficult since no simple reaction will convert the polymer into monomeric components. However, some insight into the structure of the polyethers may be obtained from examination of the simple compounds isolated from the reactions in aqueous medium. These compounds may be fragments arising from cleavage of the fluoroaryl ethers under the basic conditions of the reaction, or they may be simply products which failed to propagate into polymers.

Perhaps the most significant compound isolated was tetrafluororesorcinol. The formation of this compound suggests the direct attack of hydroxyl ion on the pentafluorophenoxide ion. It has been shown previously [5] that even strong nucleophiles such as sodamide in liquid ammonia have failed to react with pentafluorophenoxide salts. Certainly, resonance structures similar to those for phenoxide ion can be written for pentafluorophenoxide as shown below:

**Figure:**
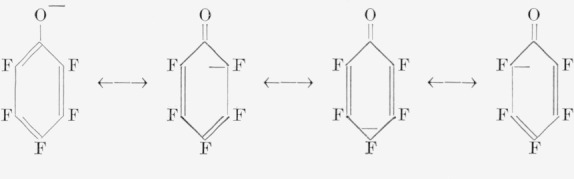


Therefore, the reaction conditions, i.e., excess base and high temperature and pressure, must have been sufficiently drastic for the incoming nucleophile to overcome the repulsion due to the negative charge of the pentafluorophenoxide. From these resonance structures the main reaction product would be expected to be the meta isomer, since these positions would be least negative and herefore vulnerable to attack. However, it was also shown that multiple replacement can occur by use of extreme conditions of base and temperature, in that only carbonlike solids were obtained at 190 °C and a 2 mole excess of potassium hydroxide. It could also be argued that tetrafluororesorcinol is the product obtained by cleavage of 1,3-bis(pentafluuorophenoxy)-tetrafluorobenzene by hydroxide ion (eq 5).

$$C_{6}F_{5}{\text{OC}}_{6}F_{4}{\text{OC}}_{6}F_{5}+2\text{KOH}\to 2C_{3}F_{5}\text{OK}+C_{6}F_{4}{\left(\text{OH}\right)}_{2}$$ (5)

In the presence of excess hydroxide it is doubtful that any of the 1,3-bis(pentafluoroplienoxy)-tetrafluuorobenzene even formed, since pentafluuorophenoxide would be a weaker nucleophile than hydroxide ion and reaction of the latter ion would be expected to take precedence over the former. The isolation of perfluuorophenyl ether and pentafluuorophenoxytetrafluorophenol, from reactions of pentafluuorophenoxide ion and hexafluorobenzene in water at high temperatures under autogenous pressure, would seem to indicate multiple modes of reaction. In the first case, pentafluorophenoxide and hexafluorobenzene react to give perfluorophenyl ether and subsequent reactions to lead to polymers (eq 6);

$$C_{6}F_{5}{\text{OC}}_{6}F_{5}+xC_{6}F_{5}\text{OK}\to C_{6}F_{5}{\left({\text{OC}}_{6}F_{4}\right)}_{x}{\text{OC}}_{6}F_{5}+x\text{KF}$$ (6)

or, the pentafluorophenoxide ion (see resonance forms) may react with itself to produce pentafluorophenoxytetrafluorophenol and eventually to polymers (eq 7)

$$2C_{6}F_{5}\text{OK}\to C_{6}F_{5}{\text{OC}}_{6}F_{5}\text{OK}\ldots$$ (7)

A third possibility also exists in that the potassium pentafluorophenoxide is a salt of a weak acid and increased amounts of free pentafluorophenol may be obtained under these conditions according to eq 8:

$$C_{6}F_{5}\text{OK}+H_{2}O\to C_{6}F_{5}\text{OH}+\text{KOH}$$ (8)

This is similar to the exchange reaction observed in the methanol reaction (eq 1). If partial hydrolysis of the potassium pentafluorophenoxide does occur at these temperatures, then reaction between the pentafluorophenoxide and the pentafluorophenol thus formed would be expected to be faster because of the reduced charge on the free phenol oxygen than on the pentafluorophenoxide ion. The feasibility of this reaction occurring has been shown by the susceptibility of pentafluorophenoxide to react easily when pentafluorophenol was used both as a solvent and a reactant.

From consideration of the various foregoing reaction sequences, the polyperfluorophenylene ethers are probably composed of both *para* and *meta* linkages (*p*>*m*). This is not too surprising since evidence was previously [5] cited that the reactions of weak nucleophiles often yield *meta* derivatives. Kornblum [22, 23] and Le Noble [24] have recently shown the effects of solvation, temperature, and pressure on the reaction course of ambident [23] ions. Similar effects would be expected to alter the reaction course of pentafluorophenoxide. Nuclear magnetic resonance spectra, which proved effective in determining simple isomers, becomes complex and difficult to interpret, even for a small compound such as pentafluorophenoxytetrafluorophenol. Although the near infrared region has been useful in eliminating the *ortho*-hydroxyl compounds from further consideration, more data are needed to distinguish clearly between the amount of *para* and *meta* linkages in these polymers.

### 7.1. Thermal Stability of Small Molecules

The thermal stability of perfluorophenyl ether was determined by the same procedure as described previously [25]. For comparative purposes individual samples of phenyl ether, biphenyl, and decafluorobenzophenone were included in the test. From 0.1 to 0.5 g of the samples were sealed in evacuated thick-walled glass capillary tubes. After 1 hour at 500 °C, the samples were examined visually. The perfluorophenyl ether appeared to be completely charred, whereas the sample of decafluorobenzophenone showed approximately 50 percent decomposition. No change was observed in the tube containing biphenyl and only a slight coloration developed in the phenyl ether sample. The perfluorophenyl ether heated at 400 °C for 1 hour showed approximately 40 percent decomposition. The relative thermal stability of the small molecules examined in the course of our work is as follows:

$$\begin{array}{r} {\left(C_{6}F_{5}\right)}_{2}\geq {\left(C_{6}H_{5}\right)}_{2}\geq C_{6}F_{6}>{\left(C_{6}H_{5}\right)}_{4}\text{Si}\geq {\left(C_{6}H_{5}\right)}_{2}O\\ >{\left(C_{6}F_{6}\right)}_{2}C=O\geq {\left(C_{6}F_{5}\right)}_{4}\text{Si}>{\left(C_{6}F_{5}\right)}_{3}P\geq {\left(C_{6}F_{5}\right)}_{2}O\\ >{\left(C_{6}H_{5}\right)}_{3}P>{\left(C_{6}F_{5}\right)}_{3}\text{PO} \end{array}$$

The results indicate that increased thermal stability may be obtained in perfluorinated aromatic polymers by incorporating C=O or CX_2_ groups between the rings instead of oxygen alone. However, the polyperfluorophenylene ethers may find considerable application where temperatures do not exceed 400 °C.

## 8. Experimental

Near-infrared spectra in the 2.5 to 3.2 *μ* range were obtained using 1 percent solutions in carbon tetrachloride. Infrared spectra of solids were obtained with pellets containing 1 to 2 mg of the compound and 300 mg of potassium bromide. Molecular weights were determined by osmometry using 1 percent solutions of the polymer in chloroform.

### 8.1. Pentafluorophenol and Tetrafluororesorcinol

A solution of 171 g (3 moles) of potassium hydroxide and 387 ml of distilled water, along with 181 g (0.97 mole) of hexafluorobenzene, were placed into an 800-ml capacity, silver-lined bomb. The bomb was rocked and heated at 150 °C for 5 hours, cooled, opened, and 139 g (77 percent) of unreacted hexafluorobenzene was recovered. The aqueous layer was acidified and extracted with two 100-ml portions of methylene chloride. After removal of the solvent by distillation there was obtained 12 g (28.8 percent) of pentafluorophenol (bp 144–145 °C) and 7 g (29.2 percent based on reacted C_6_F_6_) of tetrafluororesorcinol (bp 217–218 °C/760 mm; mp 95–96 °C). This latter compound is water soluble at 25 °C, and gives a typical blue ferric chloride test for phenols. Mass spectrometer analysis showed the parent peak at 182 mass units. Nuclear magnetic resonance data indicated the meta isomer. The melting point of the compound changes to 72–75 °C on standing in a moist atmosphere, indicating hydrate formation.

Analysis: Calculated for C_6_H_2_F_4_O_2_: C, 39.6; F, 41.7. Found: C, 40.15; F, 41.7.

When the same quantities as described above were heated at 190 °C for 20 hours, mostly carbonlike solids and very little phenolic material were obtained.

### 8.2. Perfluorophenyl Ether

#### a. Anhydrous Conditions in *N, N*-Dimethylformamide

To 6 g (0.027 mole) of potassium pentafluorophenoxide were added 14.4 g (0.077 mole) of hexafluorobenzene and 45 ml of *N,N*-dimethylformamide. No indications of reaction were observed at 25 °C for 24 hours. When heated at 70 °C for 48 hours, the color changed from yellow to dark brown. After cooling, the reaction products were poured into 200 ml of water and the aqueous layers extracted with several 50-ml portions of methylene chloride. After removal of the solvent, the residual liquid was distilled to yield 7.5 g (52 percent) of unreacted hexafluorobenzene and 2 g (25.7 percent based on reacted C_6_F_6_) of pentafluoro-*N,N*-dimethylaniline (bp 84–86 °C/1 mm; $n_{D}^{21}=1.4423$). This compound was identical to the pentafluoro-*N,N*-dimethylaniline prepared by a different method [5]. Only trace amounts of perfluorophenyl ether were formed.

#### b. Anhydrous Conditions in Tetrahydrofuran

To 21.7 g (0.098 mole) of potassium pentafluorophenoxide in a 188 ml capacity steel bomb was added 50 g (0.27 mole) of hexafluorobenzene and 25 ml of dried (LiAlH_4_) tetrahydrofuran. The bomb was rocked and heated at 120 °C for 18 hours. After cooling, the contents were poured into 100 ml of 10 percent hydrochloric acid solution, extracted with two 500-ml portions of methylene chloride, and the combined extracts dried (CaSO_4_) and distilled. There was recovered 28 g (56 percent) of hexafluorobenzene and 11 g (61 percent of pentafluorophenol. The residual liquid (1 g) solidified on cooling. Sublimation of this solid afforded two fractions: (1) mp 68–75 °C, probably a mixture of perfluorophenyl ether and pentafluorophenoxytetrafluorophenol and (2) mp 158–160 °C, appearing from analysis to be bis (pentafluorophenoxy)-tetrafluorobenzene.

Analysis: Calculated for C_18_F_14_O_2_: C, 42.05; F, 51.8. Found: C, 42.46; F, 52.4; H, 0.0.

#### c. In Methanol

Eighteen grams (0.081 mole) of potassium pentafluorophenoxide, 40 g (0.22 mole) of hexafluorobenzene, and 40 ml of methanol were rocked and heated at 120 °C for 20 hours in a 188 ml capacity bomb, cooled, and poured into 100 ml of water. The usual workup yielded 22 g (55 percent) of unreacted hexafluorobenzene and 11 g (57 percent based on reacted C_6_F_6_) of pentafluoroanisole (bp 138–139 °C). Acidification of the aqueous solution gave 6 g (40 percent) of recovered pentafluorophenol.

### 8.3. Polyperfluorophenylene Ethers From Pentafluorophenoxide Salts

#### a. Thermal Decomposition of Sodium Pentafluorophenoxide

To 9.2 g (0.05 mole) of pentafluorophenol in 50 ml of anhydrous ether was added in small pieces 1.15 g (0.05 g-atom) of sodium metal. At the end of the evolution of hydrogen, the solvent was removed in vacuo. The white sodium salt was heated for 1 hour in vacuo at 150 °C to insure complete removal of the solvent. The flask was then placed in a Wood’s metal bath and the temperature slowly raised to 345 °C at 1 mm pressure. The salt decomposed, yielding mainly a volatile solid (mp 94–150 °C) and a glassy residue. The solid, after sublimation at 120 °C and 5 mm pressure, followed by recrystallization from ligroin-toluene solution, gave white colorless plates (mp 164–165.5 °C). The material was insoluble in base, and by chemical and mass spectrometric analyses (parent peak 328 mass units) was shown to be octafluorodiphenylene dioxide.

Analysis: Calculated for C_12_F_8_O_2_: C, 43.95; F, 46.3. Found: C, 44.1; F, 44.5.

The glassy brownish residue was soluble in ether, and some purification could be effected in this manner. Analysis of the residue showed it to be polymeric in nature.

Analysis: Calculated for C_6_F_4_O: C, 43.9; F, 46.3. Found C, 44.0; F, 42.8; H, 0.3.

#### b. With Hexafluorobenzene in Water

This reaction was done in two heating steps. In the first step, 40 g (0.21 mole) of hexafluorobenzene, 12.5 g (0.189 mole) of potassium hydroxide, 0.5 g copper oxide, and 75 ml of distilled water were placed in a 188 ml capacity bomb that was rocked and heated at 175 °C for 5 hours. The second step consisted in raising the temperature to 225 °C and maintaining this temperature for 15 hours. After cooling, the contents were poured into 150 ml of water and showed only slight alkalinity to alk-acid *p*H paper. After filtration, the aqueous layer was extracted with methylene chloride. The extracts were combined with the organic layer, dried (CaSO_4_) and distilled. After removal of the unreacted hexafluorobenzene (8.1 g; 20 percent), the residue was distilled in vacuo. It was divided into a volatile liquid (3.4 g; bp 120 °C/1 mm), which on cooling solidified (mp 83–87 °C), and a nondistillable fraction. The volatile fraction (solid) was treated with aqueous sodium carbonate, and the neutral insoluble material separated by filtration. Sublimation of the filter cake showed it to contain two compounds: (1) perfluorophenyl ether (1.5 g, mp 67–69 °C) identical with the perfluorophenyl ether described previously, and (2) probably bis(pentafluorophenoxy)-tetrafluorobenzene (0.5 g, mp 154–158 °C). The aqueous carbonate solution was acidified, and the precipitate filtered, dried, and sublimed. There was obtained 1 g of pentafluorophenoxytetrafluorophenol (mp 93–94 °C). This compound was further identified by chemical, mass spectrometer (parent peak at 348 mass units) and infrared analyses (see table 1). The compound gives a ferric chloride phenol test in alcoholic solution.

Analysis: Calculated for C_12_HF_9_O_2_: C, 41.39; F., 49.3; H, 0.29. Found: C, 41.9; F, 49.4; F, 0.29.

From the nondistillable fraction there was obtained 2 g of a sublimed white solid (mp 40–85 °C) which probably contains varying amounts of the above compounds, 2.7 g of a milky grease (softening point 30 °C), and 2 g of a dark-brown brittle residue.

#### c. In the Absence of Hexafluorobenzene in Water

In a 188-ml bomb, 10 g of potassium pentafluorophenoxide and 60 ml of distilled water were rocked and heated at 200 °C for 20 hours. After cooling, the contents consisted mainly of a tarry mass. From the aqueous layer, however, 0.4 g of a white solid was separated by filtration, and purified by sublimation (mp 96–97 °C). Mass spectrometer (parent peak at 348 mass units) and infrared analyses indicate the compound to be a pentafluorophenoxytetrafluorophenol. The tar was not investigated.

#### d. In Pentafluorophenol

In a 100-ml flask, fitted with an air-cooled condenser and protected from atmospheric moisture with a drying tube, 20 g (0.109 mole) of pentafluorophenol and 2 g (0.009 mole) of potassium pentafluorophenoxide were refluxed for 3 hours. A white precipitate (potassium fluoride) began to appear after 2 hours and increased with time. At the end of 3 hours, the contents were cooled to 90 °C and distilled under vacuum. There was recovered 8 g (40 percent) of pentafluorophenol. No volatile solids were obtained at this stage. On cooling, the organic portion of the residue was dissolved in 100 ml ether and filtered from the white salts. Evaporation of the ether solution gave 10 g of a tacky, light-brown, viscous oil (I). Three and one-half grams of this oil (I) were dissolved in 5 ml of methanol, after which 1 ml of water was added and the oily layer (II) allowed to settle. The aqueous methanol layer was decanted from the oil (II) and further diluted with 2 ml of water, which precipitated an additional 2.3 g of a less viscous oil (III). Traces of solvent were removed from fraction II in vacuo by heating at 100 °C. During this treatment, 0.1 g of a white sublimate was obtained which was not further investigated. On cooling, fraction II hardened to a clear, light-brown gum and flowed freely on slight warming (40 °C). The molecular weight of fraction II was 1700.

Analysis: Calculated for C_6_F_4_O: C, 43.9; F, 46.7. Found: C, 43.6; H, 0.3; F, 41.8.

Infrared characteristics are similar to compound D as listed in table 1.

#### e. From Low Molecular Weight Polyperfluorophenylene Ethers

##### (1) From Fraction I

To 7 g of fraction I (as described above) was added 2 g of potassium pentafluorophenoxide. On heating, the salt appeared to dissolve in the oil. It was heated at 150 °C at atmospheric pressure for 2 hours, during which the contents darkened considerably. After cooling, the dark-brown, organic material was dissolved in 100 ml ether and filtered from the salts. The ether was evaporated in vacuo, and there was obtained 6 g of a dark-brown, tacky gum, which was dissolved partially in 50 ml of methanol leaving 0.5 g of insoluble material. To the methanol solution was added 5 ml of water, the oily layer was allowed to settle, and the methanolic layer was decanted. Traces of solvent were removed from the oil in vacuo at 100 °C, leaving 1.5 g of a dark-brown, brittle glass having a molecular weight of 1800. Infrared spectra still showed a free hydroxyl group in the 3600 cm^−1^ region, as well as the broad ether band at 1170 cm^−1^.

##### (2) From Fraction III

To 2.3 g of fraction III was added 10 ml of a solution containing 3 g of potassium hydroxide and 25 ml of water. The oil (III) coagulated into a milky tacky gum. The aqueous solution was decanted and the gum was dried overnight (16 hours) in vacuo at room temperature, yielding a straw colored, fluffy, easily powdered solid (1.2 g; mp 60–80 °C). The powder was degassed in a tube attached to a high-vacuum line, heated at 175 °C for 0.5 hours and then at 200 °C for an additional 0.5 hours. The material turned brown and the formation of potassium fluoride increased with time. On cooling, the contents solidified into a hard mass. Benzene (20 ml) was used to extract the polymer from the inorganic salts. A light-brown, viscous liquid was precipitated from the benzene solution with methanol (5 ml). On drying in vacuo, a light straw-colored, brittle glass (0.4 g) was obtained, very little changed in melting point. The molecular weight of this poly(fluorophenylene ether) was 4300. Since this fraction was still in the form of the potassium salt, no hydroxyl bands were observed in the infrared spectra. The spectra still showed the broad aromatic and pentafluorophenyl ether bands.

A higher molecular weight polymer (6500) was obtained by recycling the polymer through the melt polymerization again. The polymer (0.1 g) obtained in this fashion was still light-colored, more tacky, and not as brittle as the 4300 mol wt polymer.

#### f. An Insoluble Poly(perfluorophenylene Ether)

Thirteen grams of potassium pentafluorophenoxide and 2.5 g of pentafluorophenol were sealed in thick-walled, glass tube after degassing. The tube was heated for 72 hours at 200 °C. Initially, the contents became fluid at 180 °C and changed to a deep-red color. At the end of the heating period the contents no longer flowed and were dark brown in color. The tube was precooled in liquid nitrogen before opening. The contents were dissolved in 75 ml of benzene, filtered to remove potassium fluoride, and 40 ml of methanol added to the filtrate. The dark-brown precipitate was allowed to settle, the mother liquor decanted, the remaining precipitate after extraction with 25 ml of ether was a polymeric dark-brown glass (2.5 g; mp 80 °C) which flowed freely at 100 °C and had a molecular weight of 12,500. The infrared spectrum still showed the broad fluorophenyl ether band at 1170 cm^−1^. Pyrolysis gave a 50 percent weight loss after 0.5 hours at 400 °C; of the total volatiles, 20 percent were volatile at 25 °C.

Analysis: Calculated for C_6_F_4_O: C, 43.9. Found: C, 44.2; H, 0.1; K, 0.12.

From the ether-soluble fraction there was obtained 2.1 g of a polymer having a molecular weight of 3700. Characteristics of this polymer were similar to the 4300 mol wt polymer described previously.

The 12,500 mol wt fraction (2.3 g) was placed in a thick-walled tube and heated at 260 °C for 18 hours in vacuo, after which the contents no longer flowed. The tube was cooled, and the dark-brown, hard, brittle glass (2.1 g) was removed from the tube. The material was insoluble in benzene, hexafluorobenzene, ether, chloroform, acetone, and 2,5-diclilorobenzotrifluoride. The polymer softened and became rubbery at 90 °C and maintained this property up to 300 °C. Although the polymer suffered a 47 percent weight loss after 0.5 hours at 400 °C, the specimen still retained its shape but became smaller in size. As yet, the molecular weight of this polymer has not been determined.

Analysis: Calculated for C_6_F_4_O: C, 43.9. Found: C, 44.1; H, 0.1; K, 0.09.

## Figures and Tables

**Table 1:** Infrared spectral bands of some fluoroaromatic ethers and phenols in the 1300 cm^−l^ to 1100 cm*^−^*^1^ region

Compounds	Bands (cm^−1^)				
	C_6_F_5_–O–R	C_6_H_5_–O–R	OH	CF	Misc.
					
*Phenols*					
A. C_6_F_5_OH	………	………	1242(S)	1330(S)	1105(M)
B. C_6_F_4_(OH)_2_	………	………	1235(S)	1300(S)	1138(M)
C. C_6_F_5_OC_6_F_4_OH	1171(S)	………	1237(S)	1315(S)	1116(M)
D. C_6_F_5_O(C_6_F_4_O)_*x*_C_6_F_4_OH	1170(VS)	………	1237(S)	1315(S)	1117(M)
*Ethers*					
E. C_6_F_5_OC_6_F_5_	1170(S)	………	………	1310(S)	1125(W)
F. C_6_F_5_OC_6_H_5_	1169(S)	1205(VS)	………	1315(S)	1130(W)
G. (C_6_H_5_O)_2_C_6_F_4_	1168(S)	1195(VS)	………	1310(S)	1110(W)
*Fluorocarbons*					
H. C_6_F_6_	………	………	………	1300(M)	1157(M)
I. (C_6_F_5_)_2_	………	………	………	1292 (M)	1150(M)

S=strong. M = medium. W=weak.

**Table 2:** Some physical properties of PPPE

Mol. Wt.	Appearance	Soft. pt.	Sol.	Insol.
				
		(°*C*)		
1700	Amber gum	40–60	Most organic	H_2_O
3700–6500	Amber, brittle glass.	60–80	Ether, CC1_3_H, benzene.	CH_3_OH,H_2_O
12,500	Brown, brittle glass.	80–100	CCl_3_H, Benzene	Ether, CH_3_OH
X-link	Brown, brittle glass.	*90	None	………

* Temperature at which rubbery behavior appears.

## Footnotes

2 Figures in brackets indicate the literature references at the end of this paper.
